# First‐time blood donors experience less venipuncture pain than anticipated

**DOI:** 10.1111/trf.70395

**Published:** 2026-09-22

**Authors:** Johan O. Wedin, Daphne Wu, Anders E. Henriksson

**Affiliations:** ^1^ Department of Cardiothoracic Surgery and Anesthesiology Uppsala University Hospital Uppsala Sweden; ^2^ Department of Surgical Sciences Uppsala University Uppsala Sweden; ^3^ Department of Transfusion Medicine Sundsvall County Hospital Sundsvall Sweden; ^4^ Natural Sciences Mid Sweden University Sundsvall Sweden

**Keywords:** blood shortage, donor retention, pain, phlebotomy, sex differences

## Abstract

**Background:**

Little is known about the perceived pain associated with blood donation. This study aimed to investigate expected and perceived pain among first‐time blood donors.

**Study Design and Methods:**

In this prospective observational study, 140 consecutive first‐time blood donors (70 men and 70 women) were included. The donors were asked to grade the expected pain prior to their first whole blood donation and to grade the perceived pain after the procedure. The pain was graded using an 11‐point Numerical Rating Scale (NRS) from 0 (no pain) to 10 (worst imaginable pain). The association between experienced pain and drop‐out as a blood donor at 2 years was also investigated.

**Results:**

The mean age of first‐time blood donors was 30.6 years, with no difference between men and women (31.2 vs. 30.0 years, *p* = .533). First‐time blood donors significantly overestimated the venipuncture pain, with on average 1.16 units lower perceived (*β* = −1.16, *t* = −5.335, *p* < .001). Men reported both higher expected and perceived pain rates than women, but with no statistically significant interaction between pain discrepancy and sex (*β* = −0.41, *t* = −1.351, *p* = .179). In a multivariable logistic regression model, perceived pain was not associated with deregistration as a blood donor at 2 years.

**Conclusions:**

First‐time blood donors experience significantly less pain than expected while donating blood. This knowledge should be used to reduce anxiety in first‐time blood donors and profiled in donor recruitment campaigns to possibly increase the number of available blood donors.

**Abbreviation**
NRS: numerical rating scale

## 1 INTRODUCTION

Blood donation is an altruistic act that plays an essential role in improving health‐related outcomes and saving lives. While modern medicine depends on the availability of blood products, the number of available blood donors is steadily decreasing,^1^ mainly due to an aging population in combination with difficulties in recruiting new blood donors.^2^ This imbalance endangers patient safety with increased morbidity and mortality in a variety of health disorders as a result.^3^ Therefore, recruiting and retaining new blood donors is critical to maintain an adequate number of available blood donors.

Retaining new blood donors remains a significant challenge, why a higher recruitment rate is needed. When investigating barriers to blood donation among non‐donors and first‐time donors, fear of needles and fear of pain have emerged as key factors.^4^, ^5^, ^6^, ^7^, ^8^, ^9^ Fear of needles and the associated venipuncture pain is a widely recognized problem in health care in general.^10^, ^11^ Providing well‐documented knowledge regarding perceived pain is an important strategy to manage anxiety and fear of venipuncture‐related pain,^12^ especially since expectations can affect how pain is experienced.^13^ Previous reports investigating pain from different needle procedures in health care indicate that the expected pain often is higher than the perceived pain.^14^, ^15^, ^16^, ^17^, ^18^ However, no study has investigated whether the expected and perceived pain during blood donation differ. A similar pattern of lower perceived pain might also be observed among blood donors. This knowledge could be used to reduce anxiety among individuals that are hesitant to donate blood due to fear of venipuncture pain.

The aims of this study were therefore to (1) investigate if the expected and perceived pain differed in first‐time blood donors, (2) investigate if expected and perceived pain differed for men and women, and (3) investigate if the perceived pain influenced the likelihood of subsequent blood donations. We hypothesized that the expected pain was higher than the perceived pain in first‐time whole blood donors, but that no difference would be observed for men and women.

## 2 STUDY DESIGN AND METHODS

### 2.1 Participants

In this prospective observational study, 140 consecutive first‐time whole blood donors aged 18–60 years who met the general requirements for blood donation were included. In Sweden, new blood donors must first be screened for donor eligibility as they register at the blood bank. This is done by a medical questionnaire followed by blood sampling for screening tests aimed to ensure the safety of the donor (hemoglobin and ferritin levels) and future recipients (infectious diseases). The first donation cannot be made before the screening samples have been medically validated, usually a month after the first visit. When a donor is proven to be eligible, he or she receives an invitation to make the first donation. New whole blood donors eligible for their first whole blood donation were identified from the ProSang donor database (Omda AS, Oslo, Norway). Together with their invitation for whole blood donation, they received an invitation to participate in this study. All donors fulfilled the standard donation criteria. Informed consent was collected from all participants. The study protocol was approved by the regional ethics committee (Dnr 2016/434‐31) and all procedures adhered to the Declaration of Helsinki.

### 2.2 Whole blood donation and pain measurement

Whole blood donation was completed per standard operating procedures, where the venipuncture was performed by experienced phlebotomists from the antecubital fossa using a 17 Gauge siliconized needle (Fenwal Inc., IL, USA) according to WHO standards.^19^, ^20^ The donors were asked to estimate the expected pain from the procedure using the 11‐point Numerical Rating Scale (NRS), ranging from 0 (no pain) to 10 (worst imaginable pain),^21^ prior to their first whole blood donation. A total of approximately 485 mL blood was drawn from each donor (450 mL for transfusion purposes and 35 mL for mandatory screening tests), and blood donors were observed for at least 10 min after completed donation. They also received fluids and light refreshments to aid recovery. After the donation, donors were again asked to grade the perceived pain on the NRS. To minimize performance bias, donors reported the expected and perceived pain on a confidential form which was put in a sealed envelope, so the phlebotomists were not aware of their ratings. If a donor reported the pain in an interval, the mean value was used. If the first venipuncture attempt was unsatisfactory, the donors were excluded from this study. If the whole blood donation was terminated prematurely (upon either donor or staff request), the donors were asked to estimate the perceived pain afterwards and remained in the study.

### 2.3 Statistical analysis

Continuous variables are reported as mean with standard deviation, while categorical data are expressed as frequency and percentage. Differences between continuous variables were analyzed using either Student's independent *t*‐test or Student's paired *t*‐test. Differences between categorical variables were assessed with *χ* ^2^ test or Fisher's exact test, as appropriate.

For the sample size calculation, a change in NRS >1 was considered clinically significant (two‐sided 5% significance level, power = 90%). A sample size of at least 62 male and 62 female first‐time blood donors was necessary to detect differences in expected and perceived pain between sexes. To examine the overall difference between expected and perceived pain during whole blood donation, and whether men and women had different expectations and experiences, we fitted a mixed‐effects linear regression model using restricted maximum likelihood. The model included treatment group, time‐point, and their interaction as fixed effects, with random intercepts for each individual to account for repeated measures within each subject. To investigate the frequency of retained blood‐donors at 2 years after the first donation, Prosang (Omda AS, Oslo, Norway) was used. A multivariable logistic regression analysis was conducted to investigate the association between perceived pain and dropout from subsequent donations, adjusting for age, sex, and expected pain.

Statistical analyses were performed in R Statistical Software (version 4.4.2). The “lme4” package^22^ was used to fit the mixed‐effects linear regression model. A *p*‐value <.05 was considered statistically significant.

## 3 RESULTS

We included 140 first‐time blood donors in the study, of which 70 were men and 70 were women. Participant characteristics are summarized in Table 1. The mean age among first‐time blood donors was 30.6 years, with no differences between men and women. The distribution of blood groups was similar for men and women.

**TABLE 1:** Characteristics of first‐time blood donors.

	All (*n* = 140)	Men (*n* = 70)	Women (*n* = 70)	*p*
Age, years	30.6 (11.0)	31.2 (11.0)	30.0 (10.7)	.533
Blood group, *n* (%)				
A+	53 (37.9)	27	26	.678
A−	11 (7.9)	8	3	
B+	16 (11.4)	8	8	
B−	4 (2.8)	2	2	
AB+	7 (5.0)	4	3	
AB−	1 (0.7)	0	1	
0+	37 (26.4)	15	22	
0−	11 (7.9)	6	5	
Retained at 2 years, *n* (%)	119 (85.0)	62 (88.6)	57 (81.4)	.237
Time between donations, days	138 (100)	114 (50)	165 (132)	.008

Overall, the mean expected pain was significantly higher than the perceived pain (3.3 vs. 1.9, *p* < .001). A linear mixed‐effects regression model investigated differences between expected and perceived pain and whether this difference varied by sex (Figure 1). First‐time blood donors significantly overestimated the venipuncture pain, with an on average 1.16 units lower perceived pain (*β* = −1.16, *t* = −5.335, *p* < .001). Men reported both higher expected and perceived pain rates than women. However, there was no statistically significant interaction between pain discrepancy and sex (*β* = −0.41, *t* = −1.351, *p* = .179). Furthermore, pain ratings, either expected or perceived, did not vary with age (*β* = −0.003, *t* = −0.262, *p* = .793).

**FIGURE 1:**
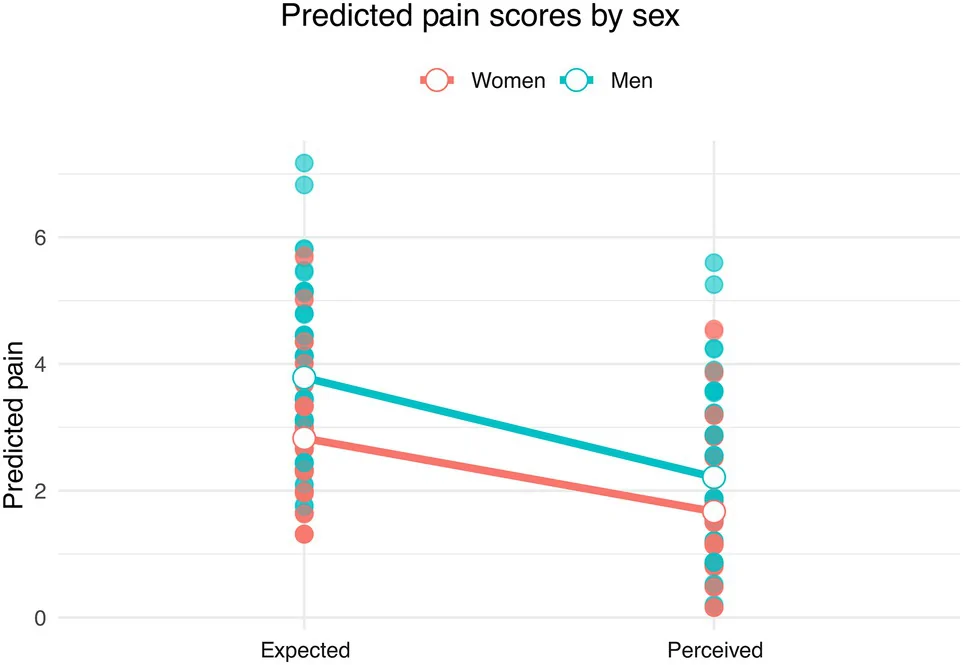
Expected and perceived pain in male and female first‐time blood donors. Both men and women reported less venipuncture pain than expected.

At the 2‐year follow‐up, 85% (*n* = 119) of the donors were still active and had made at least one additional donation while the remaining 21 donors had deregistered for various reasons. Baseline characteristics of active and deregistered donors are presented in Table 2, revealing no differences with regard to age and sex. There was a significant difference in perceived pain between blood donors that retained their status as blood donors and deregistered blood donors (1.8 vs. 2.8, *p* < .001). However, no significant association between perceived pain and deregistration was observed in neither the univariable nor the multivariable logistic regression analysis (Table 3).

**TABLE 2:** Characteristics of first‐time blood donors that were active or deregistered at 2‐year follow‐up.

	Active (*n* = 119)	Deregistered (*n* = 21)	*p*
Age, years	30.7 (10.9)	30.1 (10.5)	.820
Men, *n* (%)	62 (44.3)	8 (38.1)	.344
Expected pain	3.3 (1.9)	3.6 (2.7)	.550
Perceived pain	1.8 (1.5)	2.8 (2.2)	.049

**TABLE 3:** Univariable and multivariable logistic regression to explore association to donor retention.

	Univariable			Multivariable		
	Odds ratio	95% CI	*p*	Odds ratio	95% CI	*p*
Age, per 1‐year increase	1.00	0.95–1.04	.823	1.00	0.95–1.04	.868
Male sex	0.57	0.21–1.44	.241	0.57	0.20–1.49	.255
Expected pain	0.98	0.77–1.23	.865	1.04	0.78–1.36	.799
Perceived pain	0.94	0.69–1.24	.691	0.95	0.66–1.31	.757

## 4 DISCUSSION

Our study aimed to investigate the discrepancy between expected and perceived pain intensity among first‐time blood donors. Consistent with our a priori hypothesis, we observed a systematic overestimation of expected pain prior to blood donation, where the donors anticipated significantly higher levels than they ultimately reported after blood donation. Notably, men reported higher expected and perceived pain than women. However, the interaction between pain and sex was not significant, indicating that both men and women found the procedure less painful than anticipated.

The results of our present study align with prior research investigating expected versus perceived needle‐pain in other health‐related procedures, including venipuncture, vaccinations, dental treatment and anesthetic procedures, which consistently show that individuals tend to overestimate needle‐pain.^14^, ^15^, ^16^, ^17^, ^18^ One common reason why some individuals hesitate to donate blood is fear.^6^, ^7^, ^8^ This fear is multifactorial and not necessarily driven by concerns about complications from donating blood. Instead, fear of venipuncture pain is the most common factor to deter potential donors.^5^, ^9^ Overcoming this psychological barrier may be the most challenging aspect of deciding to donate blood and, interestingly, some individuals donate blood to address their fear of needles. It is not widely known that the antecubital fossa has fewer sensory nerve endings compared to for examples the hand, resulting in less pain perception in this area. Furthermore, since 1948, the use of relatively narrow, siliconized dispensing needles has minimized discomfort during the procedure.^20^ Experienced donors consistently report that the pain associated with blood donation is easily tolerable, a perception that has now been substantiated by our present study involving first‐time donors. Although previous studies have assessed expected and perceived pain in various contexts, our research provides the first comprehensive analysis of expected and perceived pain discrepancy in blood‐donors. Serlin et al. identified NRS = 4 and NRS = 8 as cut points, where 1–4/10 indicate mild pain, 5–8/10 indicate moderate pain, and 9–10/10 indicate severe pain.^23^ Importantly, the mean perceived pain in our study was only 2/10, showing that blood donation is associated with mild pain. On further note, although men reported slightly higher perceived pain that women which might be due to several biological and psychological factors, no sex differences in mismatch between expected and perceived pain was observed, indicating that both sexes ultimately found the procedure less painful than expected. Addressing exaggerated pain expectations through accurate, evidence‐based information could be a key strategy in recruiting new donors.

A positive blood donation experience contributes to retained blood donors and thus an adequate blood supply.^12^, ^24^ In the current study, donor retention was notably high as compared with previously reported data,^25^ with 85% of participants being active at the 2‐year follow‐up. One potential explanation behind this high retention rate could be the mild pain expectations and perceptions during the first donation. In line with this, we observed that the perceived pain was significantly higher among donors that deregistered compared to those who remained registered at 2 years. However, there was no association between perceived pain and deregistration in the logistic regression analyses. There are several possible explanations for this discrepancy, including few numbers of deregistration events (*n* = 21), with risk of type II errors. Since investigating this relationship was not the primary aim of the present study, we did not have adequate power to neither confirm nor reject such an association. We propose that this should be investigated in larger studies. If the observed pattern is confirmed in a larger, more diverse donor population, it could have practical implications for donor retention. If perceived pain is consistently associated with subsequent drop‐out, interventions aimed at reducing discomfort in first‐time donors may be considered as part of strategies to improve donor retention.

### 4.1 Strengths and limitations

There are some potential limitations to consider when interpreting the results of the present study. First, the study participants represent a selected group that had already decided to donate blood. This might impair the generalizability of our results to individuals that refrain from blood donation due to fear of needles. Second, we did not estimate anxiety, an important factor of pain adverse donation experiences.^4^, ^26^, ^27^ Hence, the anxiety among the study participants and its influence on perceived pain and subsequent donations remain unknown. A further limitation is that the present study only included individuals who had already decided to donate blood, and therefore did not capture pain expectations or factors influencing the decision to donate among individuals who had not registered to donate. This may limit the generalizability to the broader population whose pain expectations and concerns may influence their decision to donate blood. The major strengths include adequate sample size to reliably detect differences between expected and perceived pain and potential sex differences, blinded reporting, and the usage of robust and reproducible pain scale.

## 5 CONCLUSIONS

The venipuncture pain perceived by first‐time blood donors is mild and significantly lower than expected. This knowledge could be used to reduce anxiety among first‐time donors and integrated in recruitment campaigns, as it has the potential to increase the number of available blood donors.

## CONFLICT OF INTEREST STATEMENT

The authors have disclosed no conflicts of interest.

## Data Availability

The data that support the findings of this study are available on request from the corresponding author. The data are not publicly available due to privacy or ethical restrictions.
